# The Underlying Mechanism of Modulation of Transient Receptor Potential Melastatin 3 by protons

**DOI:** 10.3389/fphar.2021.632711

**Published:** 2021-02-02

**Authors:** Md Zubayer Hossain Saad, Liuruimin Xiang, Yan-Shin Liao, Leah R. Reznikov, Jianyang Du

**Affiliations:** ^1^ Department of Anatomy and Neurobiology, University of Tennessee Health Science Center, Memphis, TN, United States; ^2^ Department of Biological Sciences, University of Toledo, Toledo, OH, United States; ^3^ Program of Neuroscience, University of Maryland School of Medicine, Baltimore, MD, United States; ^4^ Department of Physiological Sciences, University of Florida, Gainesville, FL, United States; ^5^ Neuroscience Institute, University of Tennessee Health Science Center, Memphis, TN, United States

**Keywords:** transient receptor potential channels, TRPM3, protons, Pregnenolone sulfate, site-directed mutagenesis, PH sensitivity

## Abstract

Transient receptor potential melastatin 3 channel (TRPM3) is a calcium-permeable nonselective cation channel that plays an important role in modulating glucose homeostasis in the pancreatic beta cells. However, how TRPM3 is regulated under physiological and pathological conditions is poorly understood. In this study, we found that both intracellular and extracellular protons block TRPM3 through its binding sites in the pore region. We demonstrated that external protons block TRPM3 with an inhibitory pH_50_ of 5.5. whereas internal protons inhibit TRPM3 with an inhibitory pH_50_ of 6.9. We identified three titratable residues, D1059, D1062, and D1073, at the vestibule of the channel pore that contributes to pH sensitivity. The mutation of D1073Q reduced TRPM3 current by low external pH 5.5 from 62 ± 3% in wildtype to 25 ± 6.0% in D1073Q mutant. These results indicate that D1073 is essential for pH sensitivity. In addition, we found that a single mutation of D1059 or D1062 enhanced pH sensitivity. In summary, our findings identify molecular determinants respionsible for the pH regulation of TRPM3. The inhibition of TRPM3 by protons may indicate an endogenous mechanism governing TRPM3 gating and its physiological/pathological functions.

## Introduction

Transient receptor potential channels (TRP channels) are membrane proteins that allow organisms to readily sense the environment (Clapham, 2003). They detect stimuli including temperature (Brauchi and Orio, 2011; Vandewauw et al., 2018), voltage (Montell et al., 1985; Clapham, 2003; Brauchi and Orio, 2011), mechanical force (osmolarity (Strotmann et al., 2000; Liedtke and Friedman, 2003; Quallo et al., 2015), pressure (Suzuki et al., 2003), stretch (Strotmann et al., 2000; Maroto et al., 2005; Hardie and Franze, 2012), gravity (Sun et al., 2009)), light (Minke, 1977; Montell et al., 1985; Hardie, 2014), proton concentrations (Chandrashekar et al., 2006; Gerdes et al., 2007; Semtner et al., 2007), and various chemical signals (Caterina et al., 1997; McKemy et al., 2002; Everaerts et al., 2011). TRP channels form homomeric or heteromeric cation channels that can be selective or non-selective to cations; most TRP members are permeable to Ca^2+^ (Pan et al., 2011). Upon activation, TRP channels change membrane potential or intracellular calcium concentration ([Ca^2+^]_i_) to promote downstream signal transductions. Depending on sequence homology and channel architecture, TRP channels are divided into seven subfamilies, namely, TRPA (Ankyrin), TRPC (Canonical), TRPM (Melastatin), TRPML (Mucolipin), TRPP (Polycystin), TRPV (Vanilloid), and TRPN (NompC, no vertebrate member). 27 vertebrate members of these subfamilies are expressed in humans (Venkatachalam and Montell, 2007; Uchida et al., 2019).

TRPM3 belongs to the subfamily of TRPM (Melastatin). TRPM3 is a non-selective cation channel that is permeable to Ca^2+^, Na^+^, Mn^2+,^ and Mg^2+^ ions with a permeability ratio of P_Ca_/P_Na_ of 1.57 ± 0.31 (Grimm et al., 2003). RT-qPCR analyses have shown expression of human (hTRPM3), mouse (mTRPM3), and rat (rTRPM3) TRPM3 in a variety of tissues, with the most abundant expression in the brain, kidney, pituitary gland, and adipose tissues (Zamudio-Bulcock et al., 2011; Oberwinkler and Philipp, 2014). In sensory neurons, TRPM3 functions as a noxious heat sensor. For example, TRPM3 deficient mice lack the normal response to noxious heat and do not develop inflammatory heat hyperalgesia (Vriens et al., 2011; Vandewauw et al., 2018). Vangeel et al., also recently demonstrated functional expression of TRPM3 in a large subset of nociceptor neurons in humans, Thus, TRPM3 has been suggested as a potential drug target for novel analgesics (Moran and Szallasi, 2018; Vangeel et al., 2020). Although the role of TRPM3 in the central nervous system has not been explored in detail yet, a recent study showed high-level expression of TRPM3 in mouse CA2 and CA3 hippocampal neurons and in the dentate gyrus. Field potential recordings suggested that TRPM3 agonists inhibit synaptic transmission, and reduce long-term potentiation (LTP) in the mouse hippocampus (Held et al., 2020). In humans, genetic analyses have linked TRPM3 mutations with intellectual disability, epilepsy, inherited cataracts, and glaucoma (Bennett et al., 2014; Dyment et al., 2019). In the cardiovascular system, TRPM3 localizes in the perivascular nerves of mouse mesenteric arteries and induces vasodilation by stimulating CGRP (calcitonin gene-related peptide) receptors (Alonso-Carbajo et al., 2019). TRPM3 forms homomultimeric channels, which are constitutively active (Grimm et al., 2003). However, TRPM3 can also be activated by the endogenous neurosteroid pregnenolone sulfate (PS), as well as nifedipineand clotrimazole. Mefenamic acid, diclofenac, progesterone, and flavanones have been reported to inhibit TRPM3 (Wagner et al., 2008; Uchida et al., 2019). PS has been shown to increase neurotransmitter release, strengthen synaptic transmission, and modulate synaptic plasticity (Smith et al., 2014). However, whether TRPM3 contributes to any of these neuronal functions of PS or not, is not clear (Zamudio-Bulcock et al., 2011). One study has shown that the PS-induced potentiation of spontaneous glutamate release in Purkinje neurons of developing rats is mediated by TRPM3 (Zamudio-Bulcock et al., 2011). In non-neuronal cells, PS upregulates activator protein 1 (AP-1) and early growth response protein 1 (Egr-1) transcriptional activity, which can be blocked by TRPM3 antagonists (Lesch et al., 2014). TRPM3 also upregulates c-Jun and c-Fos promoter activity and stimulates CRE-controlled reporter gene transcription in insulinoma and pancreatic β-cells, in a TRPM3-dependent manner (Muller et al., 2011). In vascular smooth muscle cells, PS increases [Ca^2+^]_i_ and modulates contractile responses, which can be inhibited by TRPM3 inhibitors (Naylor et al., 2010). PS also increases [Ca^2+^]_i_ in fibroblast-like synoviocytes and suppresses the secretion of hyaluronan via TRPM3 (Ciurtin et al., 2010).

Extracellular and intracellular protons modulate ion channel activity. Indeed, intracellular acidosis activates inward rectifier K^+^ channel family protein Kir6.1, K2p channel TREK-1, and TREK-2. However, the activity of Kir1.1, Kir4.1, and Kir5.1 is inhibited by both intracellular and extracellular protons. Gap junction channels, connexins, are also inhibited by intracellular acidosis. In addition, both intracellular and extracellular acidification block the K2p channel TRESK, as well as depress or inhibit TWIK-1 and TWIK-2. Some of the ion channels, such as, TASK-2, TRESK, TALK-1, and TALK-2, which are inhibited at low pH can be activated by alkalization. Protons cannot activate or inhibit P2X2 and P2X5 homomultimers but decrease the potency and efficacy of ATP gating of P2X5 and sensitize P2X2 receptors to ATP. TRP channels are no exception regarding variable activity in response to pH. Specifically, TRPV1, TRPV4, and TRPC4 are activated by a reduction of pH. In contrast, TRPC5 currents are increased by an acidic pH until 6.0, at which point further decreases in pH reduce current (Holzer, 2009). PKD2L1 (TRPP2) expressing neurons show action potentials in response to citric acid (Huang et al., 2006), whereas intracellular and extracellular pH inhibits TRPM2 (Du et al., 2009; Starkus et al., 2010; Yang et al., 2010). Yet very little is known about how pH regulates TRPM3. In the current study, we examined how TRPM3 activation by PS is modulated by different extracellular and intracellular pH conditions. In all experiments, we activated TRPM3 by external application of PS. Thus, all subsequent mentions of TRPM3 activity in this manuscript must be considered as TRPM3 activity in response to PS, and not TRPM3 constitutive activity. As PS-induced TRPM3 currents are almost two orders of magnitude higher than TRPM3 constitutive currents (Grimm et al., 2003; Lee et al., 2003; Wagner et al., 2008), we concluded that for our experiments, it is reasonable to exclude the effects of constitutive TRPM3 activity.

## Materials and Methods

### Plasmid and Molecular Biology

The cDNA of the human TRPM3 channel (accession number AJ505026) with C-terminal GFP tag was provided by Christian Harteneck (University of Tübingen, Tübingen, Germany) (Grimm et al., 2003). Alternative splicing patterns of TRPM3 is highly conserved across human and rodents (Oberwinkler et al., 2005). To date, 25 isoforms of mTRPM3 protein have been identified, including a recently discovered variant - TRPM3γ3. Splicing events affect exons 8, 13, 15, 17, 20, 24, and 28 of TRPM3. α variants lack exon 2; β variants lack exon 1; and γ variants lack a large part of exon 28 (Oberwinkler and Philipp, 2014; Uchida et al., 2019). Our hTRPM3 cDNA contains all 30 exons, where 389 amino acids in exon 28 have been replaced with the alternative carboxy terminus of seven residues.This truncation does not affect functional activity of the ion channel (Grimm et al., 2003; Oberwinkler and Philipp, 2014). Mutations of hTRPM3-GFP were generated by site-directed mutagenesis (performed by GENEWIZ Inc.). The predicted mutations were verified by sequencing analysis.

### Cell Culture and Overexpression of hTransient Receptor Potential Melastatin 3 Channel-GFP and the Mutants in HEK-293 Cells

Human embryonic kidney (HEK)-293 cells were used to transiently overexpress wild-type hTRPM3-GFP and its mutants. The cells were grown in DMEM/F12 medium (Fisher Scientific, Waltham, MA, United States. Catalog no. MT10090CV) supplemented with 10% bovine growth serum (HyClone, Logan, UT, United States. Catalog no. SH30541.03), 100 U/ml penicillin/100 mg/ml streptomycin (Fisher Scientific, catalog no. SV30010) at 37°C in a 5% CO_2_, humidity-controlled incubator. Lipofectamine 2000 (Thermo Fisher Scientific, Waltham, MA, United States. Catalog no. 18324012) was used for the transfection of TRPM3 into the cells in a 35 mm culture dish according to the manufacturer’s instructions. Successfully transfected cells were identified by their fused GFP when illuminated at 480 nm excitation wavelength. Electrophysiological recordings were conducted between 36- and 48-h post-transfection.

### Electrophysiology

All patch-clamp experiments were performed at room temperature (20–22°C). TRPM3 whole-cell currents were recorded using an Axopatch 200B amplifier. Data were digitized at 10 kHz and digitally filtered offline at 5 kHz. Patch electrodes were pulled by Sutter P-97 micropipette puller and fire-polished to the resistance of 3–5 MΩ when filled with internal solutions. Series resistance (Rs) was compensated up to 90% to reduce series resistance errors to <5 mV. Cells in which Rs was >8 MΩ were discarded (Du et al., 2009).

For whole-cell current recording, ramp voltage stimuli (250 ms duration) were delivered at 1 s intervals and ranging from −100 to +100 mV. The internal pipette solution for whole-cell current recordings contained (in mM): 115 Cs-methanesulfonate (CsSO_3_CH_3_), 8 NaCl, 10 Cs-EGTA, 5 Na_2_-ATP and 10 HEPES, with pH adjusted to 7.2 with CsOH. In high intracellular Ca^2+^ experiments, 0.93 mM CaCl_2_ was added to the above-mentioned intracellular solution and EGTA was reduced to 1 mM, resulting in 1 µM free intracellular Ca^2+^. MaxChelator (https://somapp.ucdmc.ucdavis.edu/pharmacology/bers/maxchelator/downloads.htm) software from the University of California, Davis was used to calculate free [Ca^2+^]_i_. To avoid proton activated Cl^−^ currents conducted by endogenous anion channels of HEK-293 cells (Lambert and Oberwinkler, 2005), NaCl in standard Tyrode solution was replaced with Na-glutamate for all whole-cell current recordings. This external solution contained (in mM): 145 Na-glutamate, 5 KCl, 2 CaCl_2_, 1 MgCl_2_, 10 HEPES, and 10 glucose, with pH adjusted to 7.4 with glutamic acid. Internal and external acidic pH solutions were prepared as described previously with slight modifications (Du et al., 2009). In brief, 10 mM HEPES was replaced by 10 mM MES for the solutions at pH ≤ 6.0. Bath solutions containing 1 mM–60 mM NH_4_Cl were prepared by decreasing Na^+^ concentrations to 85 mM to keep the osmolarity constant Osmolarity was adjusted to 300 ± 10 mOsm with mannitol. In experiments designed to test protons permeability of TRPM3, pipette solutions contained (in mM): 120 NMDG, 108 glutamic acid, 10 HEPES, 10 EGTA, with pH adjusted to 7.2 with NMDG. External solutions for testing proton permeability contained (in mM): 145 NMDG, 10 HEPES, and 10 Glucose; and pH was adjusted with glutamic acid. To prepare the pH 5.5 external solution for proton permeability, 10 mM HEPES was replaced with 10 mM MES. PS was dissolved in DMSO to prepare 100 mM stock solution, and an adequate volume of stock PS solution was added to the external solution to achieve the required concentration. All the chemicals used in electrophysiological experiments were from Sigma-Aldrich, St. Louis, MO, United States.

### Data Analysis

Statistical data were analyzed using GraphPad Prism 8. Pooled data are presented as mean ± SEM. Concentration-response curves were fitted by an equation of the form: *E = E*_*max*_{1/[1+(EC_50_/C)^n^]} where *E* is the effect at concentration *C*, *E*_*max*_ is the maximal effect, IC_50_ is the concentration for half-maximal effect, and n is the Hill coefficient (Du et al., 2009). The concentration of proton required for half-maximal inhibition is denoted by IC_50_ (when proton concentration is expressed as molar concentration) and pH_50_ (when proton concentration is expressed by pH value). Statistical comparison of two groups was performed by unpaired Student’s t-test, with *p* < 0.05 considered statistically significant. Statistical comparison of three or more groups was performed by one-way ANOVA with Tukey’s post hoc multiple comparisons.

## Results

### Extracellular and Intracellular Acidic pH Inhibits Transient Receptor Potential Melastatin 3 Channel

**FIGURE 1:**
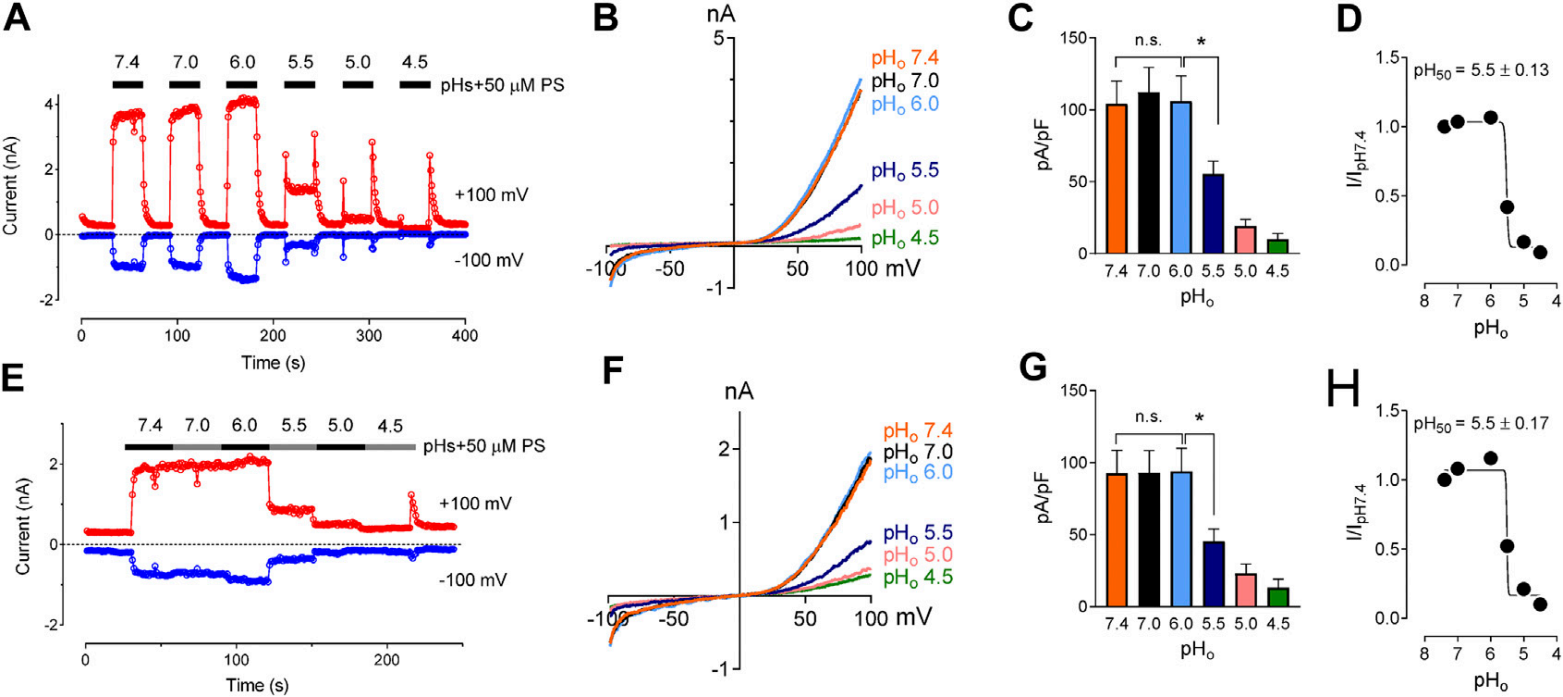
Inhibitory effect of extracellular acidic pH on TRPM3 activation by PS. **(A)** Time course of TRPM3 currents elicited by voltage ramps ranging −100 to +100 mV. Both inward and outward currents were completely and reversibly inhibited by pH_o_ 4.5 50 µM PS was applied extracellularly and was washed with PS-free extracellular solution between subsequent PS application. Inward and outward currents were measured at −100 and +100 mV, respectively. **(B)** Representative recording of TRPM3 current in **(A)** by ramp protocols ranging from −100 to +100 mV at the indicated pH_o_. **(C)** Mean current amplitude of TRPM3 at the indicated pH_o_ in **(A)** (mean ± SEM; *n* = 11, * indicates *p* < 0.05 by unpaired Student’s t-test; “n.s.” indicates not statistically significant). **(D)** pHo concentration-dependence of TRPM3 activation by PS. TRPM3 currents exerted at +100 mV were utilized for the plot. The current amplitude at the indicated pH_o_ normalized to the current amplitude at pH_o_ 7.4 in **(A)**. Background electrical activity before application of PS is subtracted in all quantitative analysis. **(E)** Time course of TRPM3 currents elicited by voltage ramps ranging −100 to +100 mV. PS was applied continuously while reducing extracellular pH without allowing any washing period between subsequent extracellular solution applications. **(F)** Representative recording of TRPM3 current in **(E)** by ramp protocols ranging from −100 to +100 mV at the indicated pH_o_. **(G)** Mean current amplitude of TRPM3 at the indicated pH_o_ in **(E)** (mean ± SEM; *n* = 7, * indicates *p* < 0.05 by unpaired Student’s t-test; “n.s.” indicates not statistically significant). **(H)** pH_o_ concentration-dependence of TRPM3 activation by PS. TRPM3 currents exerted at +100 mV were utilized for the plot. The current amplitude at the indicated pH_o_ normalized to the current amplitude at pHo 7.4 in **(E)**.

**FIGURE 2:**
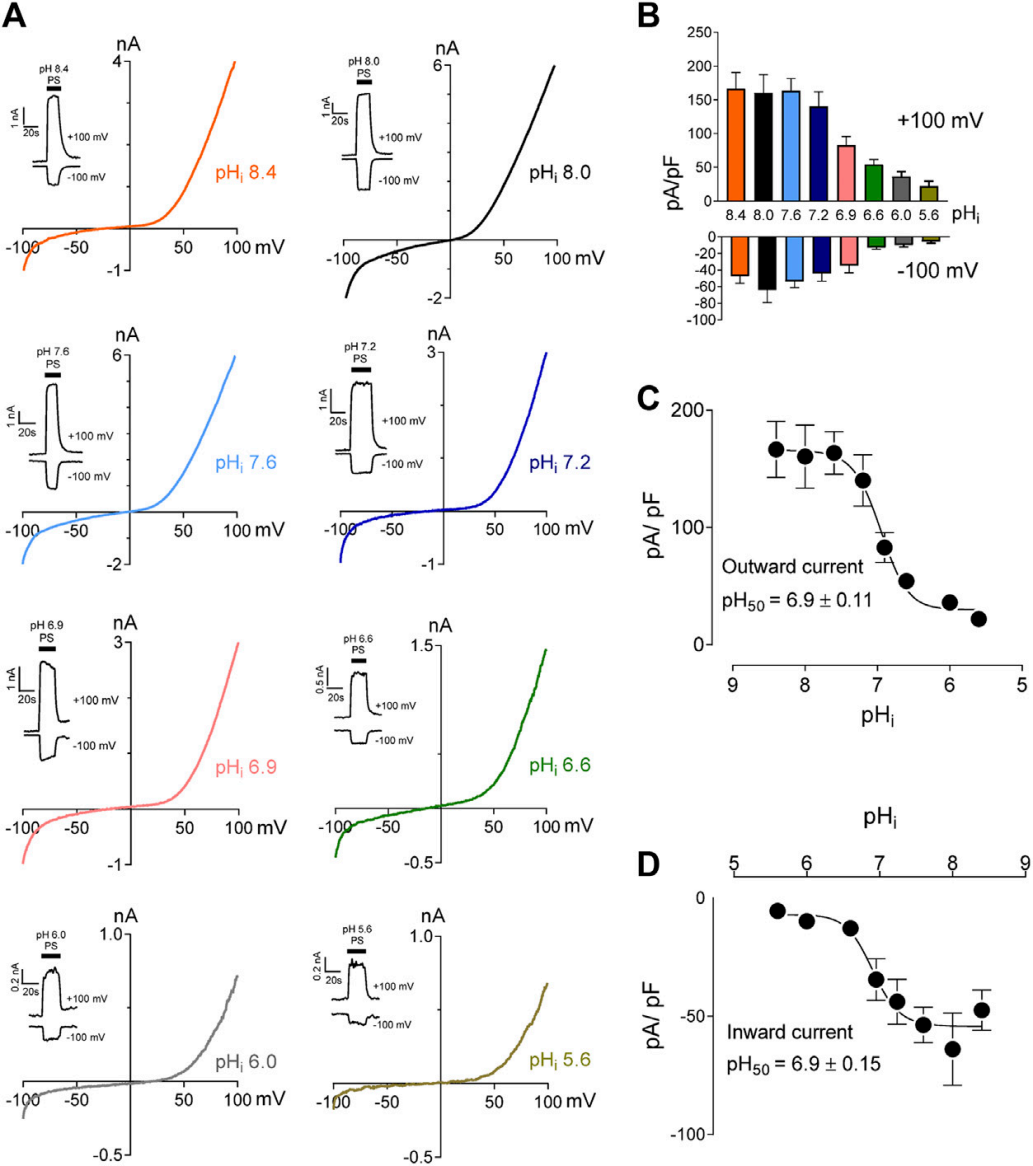
Intracellular acidification blocks TRPM3 activation by PS in a concentration-dependent manner. **(A)** Representative recordings and time courses (insert) of TRPM3 current by ramp protocols ranging from −100 to +100 mV at the indicated pH_i_. PS was applied through the extracellular solution (pH_o_ 7.4) and different cells were exposed to different pH_i_ while keeping that pH_i_ constant. **(B)** Mean current amplitude of TRPM3 at the indicated pH_i_ (mean ± SEM; *n* = 8–14). **(C,D)** pH_i_ concentration-dependence of TRPM3 activation by PS. c and d show outward and inward current, respectively, elicited by TRPM3 after extracellular PS application, by voltage ramp ranging −100 to +100 mV. TRPM3 currents exerted at −100 and +100 mV were considered as inward and outward currents, respectively, and were utilized for these plots. All currents were normalized to the corresponding capacitance of the cell overexpressing hTRPM3-GFP. Each data point is the mean of 8–14 cells with the error bar showing SEM, at the indicated pH_i_. Inhibitory pH_50_ values were measured separately for outward (pH_50_ = 6.9 ± 0.11) and inward (pH_50_ = 6.9 ± 0.15) currents.

We studied the effects of low pH on TRPM3 by overexpressing hTRPM3-GFP in HEK-293 cells and recording whole-cell currents in response to PS. We found that low extracellular pH inhibited TRPM3 in a reversible manner (Figures 1A–C). To avoid proton activated endogenous anion channel conducted Cl^−^ currents, external Cl^−^ was replaced by glutamate (See *Methods*). The inhibitory effect of low extracellular pH (pH_o_) was only observed below pH 6.0. Specifically, at pH_o_ 7.0 and 6.0, recorded TRPM3 currents were equivalent to pH_o_ 7.4 (*p* > 0.05 in both groups). At a pH_o_ below 6.0, acidic conditions exhibited significant inhibition of TRPM3. pH_o_ 5.5, caused ∼60% reduction in TRPM3 whole-cell current induced by PS (Figure 1). Although the concentration-dependent effect of extracellular pH on TRPM3 activity was weak, fitting these data in a non-linear regression curve resulted in a concentration-dependent relationship with a pH_50_ value of 5.5 ± 0.13, and, Hill coefficient of n_H_ = 2.5 (Figure 1D). When PS was applied continuously while reducing extracellular pH (Figures 1E–G), a concentration-dependent relationship was observed with a pH_50_ value of 5.5 ± 0.17, and a Hill coefficient of n_H_ = 2.6 (Figure 1H). To further measure the inhibitory effect of low extracellular pH on TRPM3, we held the membrane potential at +60 mV continuesly during the recordings. We found that low pH significantly blocked PS-induced TRPM3 currents (Supplementary Figure S1). Interstingly, as shown in Figure 1A, application of low extracellular pH (pH ≤ 5.5) with PS produced an initial activation of TRPM3 before blocking it. This suggested that the onset of low pH_o_ inhibition is slower than the PS activation. Because acidic pH has been shown to block TRPM channels intracellularly (Du et al., 2009; Starkus et al., 2010; Yang et al., 2010), we next asked if intracellular low pH could inhibit TRPM3. To answer this question, we first investigated the effects of intracellular low pH (pH_i_) on TRPM3. Whole-cell TRPM3 currents were recorded using the low-pH pipette solutions (see experimental procedures) while keeping extracellular pH constant at 7.4 (Figure 2A). To investigate the extent of modulation of TRPM3 by protons, we also introduced higher pH_i_ than the physiological pH_i_ of 7.2. we found that the TRPM3 current plateaued at about pH_i_ 7.6. Acidic pH had similar inhibitory effects on both the outward and inward TRPM3 currents (Figure 2A). There was no significant difference in the steady-state inhibition between inward and outward currents, suggesting that there were no voltage-dependent effects of acidic pH_i_ on inward and outward TRPM3 currents (Figure 2B). Low pH_i_ markedly reduced TRPM3 current in a concentration-dependent manner with a pH_50_ value of 6.90 ± 0.11 (outward current at +100 mV) (Figure 2C) and pH_50_ value of 6.90 ± 0.15 (inward current at −100 mV) (Figure 2D). The Hill coefficient was n_H_ = 1.6. Combined, our findings suggesting that extracellular acidic pH 6.0 did not affect TRPM3 current but low intracellular low pH had an inhibitory pH_50_ value of 6.9. Thus, these data suggest that TRPM3 sensitivity to pH was stronger on the cytoplasmic side.

### Effect of Low pH_i_ on Concentration-Dependence of Transient Receptor Potential Melastatin 3 Channel Activation by Pregnenolone Sulfate

**FIGURE 3:**
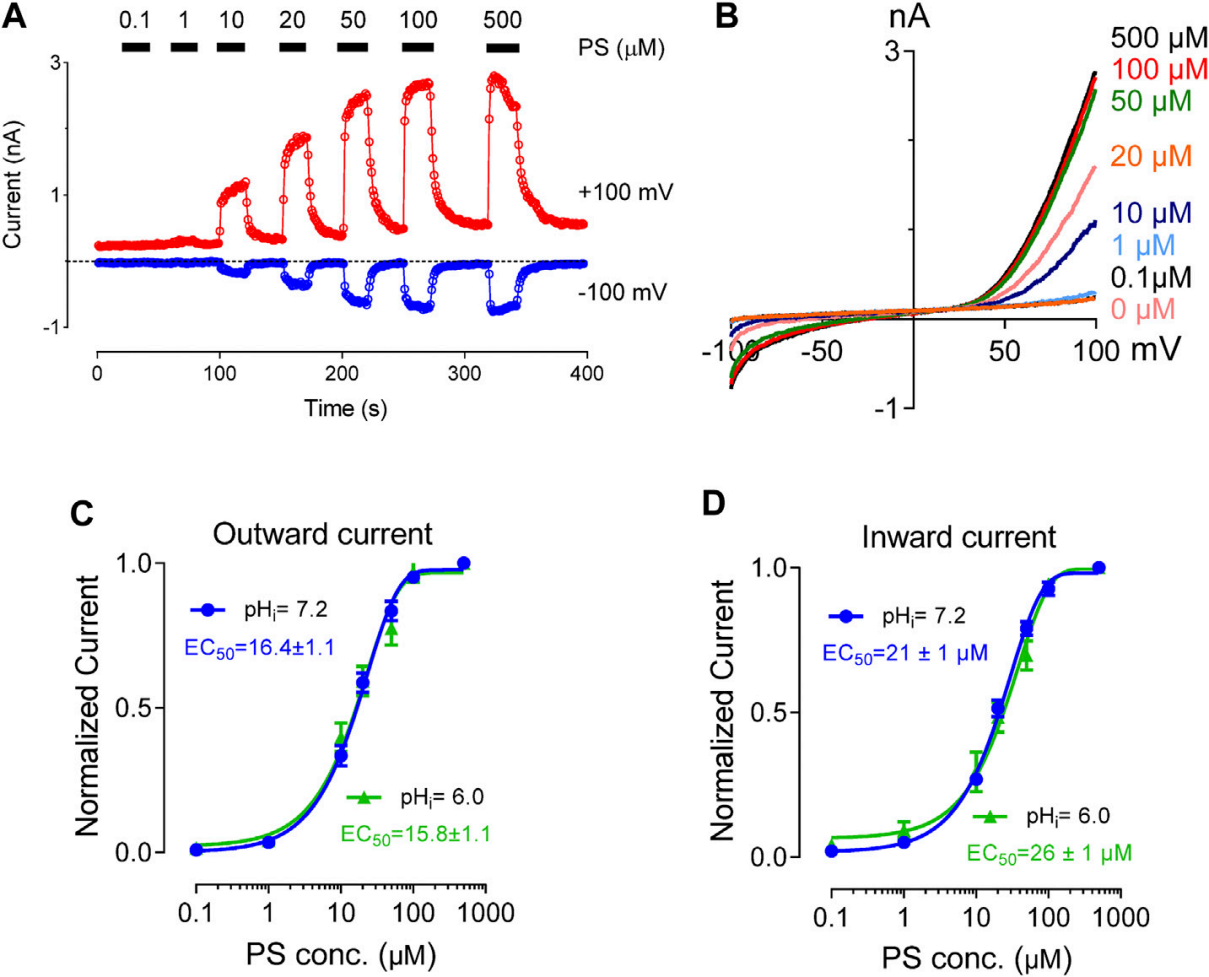
Concentration-response curve for PS-induced currents in hTRPM3, at the indicated intracellular pH. **(A)** Time course of TRPM3 currents elicited by voltage ramps ranging −100 to +100 mV. PS was applied extracellularly in increasing concentration sequence at concentrations of 0, 0.1, 1, 10, 20, 50, 100, and 500 µM, with an adequate washing period between subsequent PS applications. **(B)** Representative recording of TRPM3 current at the indicated PS concentration in **(A)**. **(C)** Outward current normalized to maximum concentration-response (500 µM) of the PS, with error bars showing SEM (*n* = 7 cells). The EC_50_ values of PS for the outward currents are 16.4 ± 1.1 µM at pH 7.2 and 15.8 ± 1.1 µM at pH 6.0. **(D)**. Inward current normalized to maximum concentration-response (500 µM) of the PS, with error bars showing SEM (*n* = 7 cells). The EC_50_ values of PS for the inward currents are 21.0 ± 1.0 µM at pH 7.2 and 26.0 ± 1.0 µM at pH 6.0.

PS binds directly to the extracellular side of the TRPM3 channel to activate it. TRPM3 channel stays both functional and unaffected by the presence of intracellular PS (Wagner et al., 2008). Previous studies have shown that PS activates TRPM3 in a concentration-dependent manner with an EC_50_ value of 12 and 23 µM for outward and inward current respectively (Wagner et al., 2008). To investigate the effect of protons on PS concentration-dependent activation of TRPM3, we perfused the cells with a wide range of PS concentrations (1–500 µM). pH_i_ was held constant at 7.2 or 6.0. TRPM3 whole-cell outward and inward currents showed very similar PS concentration-dependence in both pH_i_ conditions (Figures 3A,B). The EC_50_ values for the outward currents were 16 µM (pH_i_ 7.2) and 15 µM (pH_i_ 6.0) (Figure 3C). This result suggested that higher proton concentrations inside the cell decreased TRPM3 maximal activation at any given PS concentration. However, protons did not affect the PS concentration-dependent activation of TRPM3 as EC_50_ values for the inward currents were 21 µM (pH_i_ 7.2) and 26 µM (pH_i_ 6.0) (Figure 3D). This result indicated that the PS concentration-dependent curve of TRPM3 inward currents did not show any change in response to low pH_i_. In summary, these data suggested that intracellular protons do not compete with PS for binding sites.

### Transient Receptor Potential Melastatin 3 Channel Inhibition by Protons can be Reversed by Increasing pH_i_

**FIGURE 4:**
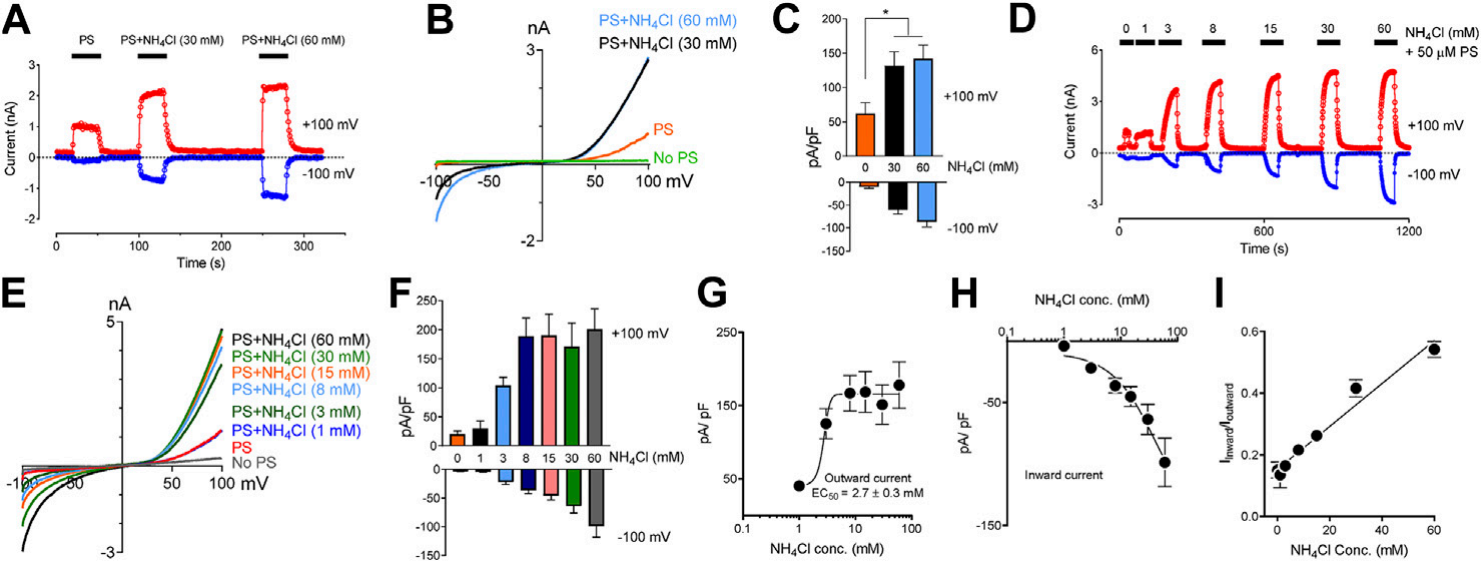
Inhibitory effect of low intracellular pH on TRPM3 can be reversed by perfusing cells with extracellular solution containing NH_4_Cl. **(A)** Time course showing outward and inward current, recorded at +100 and −100 mV respectively, obtained from HEK cells overexpressing TRPM3, under voltage ramp protocol ranging −100 to +100 mV, (pH_i_ = 6.0, *n* = 11 cells). Indicated concentrations of NH_4_Cl was applied extracellularly along with PS. To achieve similar osmolarity, all extracellular Na^+^ concentration was lowered to 85 mM and osmolarities were adjusted to 300 ± 10 mOsm by mannitol. Adequate washing time (1–3 min) was provided after each application of NH_4_Cl, to bring the current down to the basal level, which is 6.0 for all the cells recorded. Representative current was plotted vs. time in the presence of extracellular NH_4_Cl and PS, at the indicated concentrations. To achieve similar osmolarity, all extracellular buffer Na^+^ concentration was lowered to 85 mM and osmolarities were adjusted to 300 ± 10 mOsm by mannitol. **(B)** Representative recording of TRPM3 current by ramp protocols ranging from −100 mV to +100 mV at the indicated NH_4_Cl concentrations. **(C)** Mean outward and inward TRPM3 current at the indicated NH_4_Cl concentrations (mean ± SEM, *n* = 11 cells). * indicates *p* < 0.05 by unpaired Student’s t-test. **(D)** Representative recording of TRPM3 current by the dose-dependent effects of NH_4_Cl. The applications of extracellular NH_4_Cl at the indicated concentrations. **(E)** Representative recording of TRPM3 current by ramp protocols ranging from −100 mV to +100 mV at the indicated NH_4_Cl concentration. (From panel d) **(F)** Mean outward and inward TRPM3 current at the indicated NH_4_Cl concentration. (From replicated experiment of **(D)**) (mean ± SEM, *n* = 11 cells). **(G,H)** Concentration-dependence curves of effects of NH_4_Cl on TRPM3 activations, outward **(G)** and inward **(H)** currents, respectively. All currents were normalized to the corresponding capacitance of the cell overexpressing hTRPM3 (mean ± SEM, *n* = 11 cells) at the indicated NH_4_Cl concentration. EC_50_ values were measured separately for outward (NH_4_Cl_50_ = 2.7 ± 0.3 µM) currents, whereas the inward currents continue to increase after applying 60 mM NH_4_Cl. **(I)** Mean ratio of inward current to outward current plotted against NH_4_Cl concentrations (mean ± SEM, *n* = 11 cells).

Extracellular application of NH_4_Cl produces a rise in pH_i_ resulting from an influx of NH_3_. NH_3_ binds intracellular protons and causes alkalization inside the cells (Jacobs, 1922; Warburg, 1922). We observed that protons blocked TRPM3 from the cytoplasmic side. Thus, we tested whether raising pH_i_ by applying NH_4_Cl could rescue the TRPM3 currents. We perfused the cells with solutions where part of NaCl was replaced by an equal amount of NH_4_Cl (see experimental procedures), to test whether increasing pH_i_ while keeping extracellular pH the same can reverse the blocking effects of protons on TRPM3. As shown in Figure 4A,B, 30 and 60 mM NH_4_Cl potentiated the PS-induced TRPM3 currents significantly, while potentiation was only slightly different on the outward and inward currents (Figure 4C) We attribute this increase to the influx of NH_3_ into the cell and removing bound protons from the TRPM3 cytoplasmic side. Overall, this result supported our finding that TRPM3 could be blocked by an acidic intracellularpH Indeed, different concentrations of NH_4_Cl were applied to the same cell, and all NH_4_Cl applications changed intracellular pH. To minimize the residual effects of NH_3_, we allowed adequate washing time between two consecutive NH_4_Cl + PS applications (Figure 4D–F).

Subsequently, we tested whether external NH_4_Cl exhibited a concentration-dependent rescuing effect on TRPM3 activity or not. We perfused the cells with modified Tyrode solutions containing different concentrations of NH_4_Cl (1–60 mM) (Figure 4D–F). Na^+^ concentrations in all solutions were kept the same, and osmolarities were adjusted to 300 ± 10 mOSM with mannitol. We observed a five-fold increase in TRPM3 activity at only 3 mM (NH_4_Cl) concentration (Figure 4D). Even 1 mM NH_4_Cl rescued some TRPM3 activity (*p* < 0.05) (Figure 4D). Although, when compared with 3 mM NH_4_Cl, some of the higher concentrations showed a significant increase in TRPM3 activity, there was no substantial increase in TRPM3 activity by further increasing the NH_4_Cl concentration. The PS concentration-dependent manner with a EC_50_ value of 2.7 ± 0.3 µM, the Hill coefficient is nH = 1.1 for the outward currents (Figure 1G) and inward currents (Figure 1H). We also observed a higher increase in inward current than corresponding outward current (Figure 4I), with the ratio of inward current to the outward current increasing from 0.13 at 1 mM NH_4_Cl to 0.54 at 60 mM NH_4_Cl. When plotting the correlation between the outward and inward currents, we observed a linear realationship between the outward and inward currents (Figure 1I). It is important to note that because we did not measure the exact pH_i_ changes following external NH_4_Cl applications, the pH_i_ differences before and after NH_4_Cl applications, are unknown. However, despite this limitation, these experiments support the claim that TRPM3 activity is highly sensitive to changes in intracellular pH changes in the physiological pH_i_ range (pH_i_ 6.0–8.0). ITo ensure that application of NH_4_Cl and PS for an extended period of time did not affect TRPM3 activity and thus confound our results, we also examined individual cells following only one exposure to different concentrations of NH_4_Cl. The results of these experiments are summarized in Supplementary Figure S2A. Perfusion of individual cells with different concentrations of NH_4_Cl (Supplementary Figure S2B) showed similar response when compared to TRPM3 currents from our earlier experiments (Figure 4D). In summary, these data suggest that the increase of intracellular pH boosts TRPM3 activities.

### Transient Receptor Potential Melastatin 3 Channel is Potentially Permeable to Protons

**FIGURE 5:**
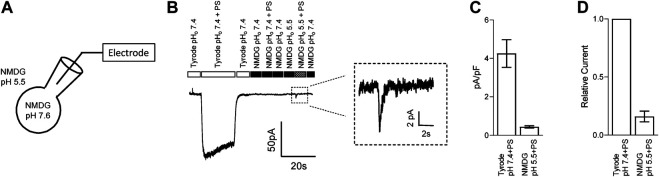
TRPM3 is potentially permeable to protons. **(A)** Schematic showing the protons permeation recording condition, where all intracellular and extracellular ions were replaced by NMDG and glutamic acid, respectively, except for protons. 50 µM PS was applied while holding hTRPM3-GFP transfected HEK cells at −100 mV. **(B)** Inward currents were elicited by the PS application. No PS activated current under the NMDG solution at pH_o_ 7.40, while lowering down the pH_o_ to 5.50 generated a small and transient inward current. Insert panel shows the inward current recorded during NMDG-PS (pH 5.5) application. **(C)** Mean current amplitude in response to PS at the indicated conditions (mean ± SEM, *n* = 8 cells). **(D)** Relative current amplitude in response to PS, comparing with Tyrode (pH_o_ 7.4) solution response of the same cell (mean ± SEM, *n* = 8 cells).

The lipid bilayer (*eg*., cell membrane) presents a strong barrier for the transport of charged ions through eukaryotic cell membranes. Although the permeability of protons is higher than other monovalent cations (Tepper and Voth, 2005), it is unlikely that these mechanisms can transport sufficient protons across the membrane to have a direct impact unless protons are passing through the overexpressed ion-channel itself. We thus examined whether protons can permeate through TRPM3. To test this, we recorded TRPM3 inward current in HEK-293 cells, overexpressing hTRPM3-GFP, by holding the membrane at −100 mV and applying PS and low pH solutions externally. We maintained pH_i_ at 7.6 to provide a higher concentration gradient for protons. All cations except protons were removed by using NMDG in the external and internal solutions (Figure 5A). PS, along with NMDG pH 5.5 extracellular solution, produced a small transient inward current in TRPM3 transfected HEK-293 cells (Figure 5B). Indeed, the amplitude of this current was significantly lower than the TRPM3 currents observed using theTyrode solution (Figures 5C,D). This result was likely due to removal of all cations besides a limited amount of protons, with the extracellular and intracellular proton concentrations calculated to be 3.2 × 10^–3^ mM (pH 5.5) and 2.5 ×10^–5^ mM (pH 7.6), respectively. These cation concentrations are several orders of magnitude lower than the total cation concentrations of the Tyrode solution. However, since there were no other cations in our solution and although low in amplitude, the current we observed suggests the passage of proton through the TRPM3 channel. We spectulate that the transient nature of the current is because the intracellular protons inhibit TRPM3 after their permeation. Mock transfected cells did not produce any inward current in response to PS (Data not shown). This evidence indicates that TRPM3 is potentially permeable to protons.

### The Inhibition of Intracellular Ca^2+^ on Transient Receptor Potential Melastatin 3 Channel is pH-Independent

**FIGURE 6:**
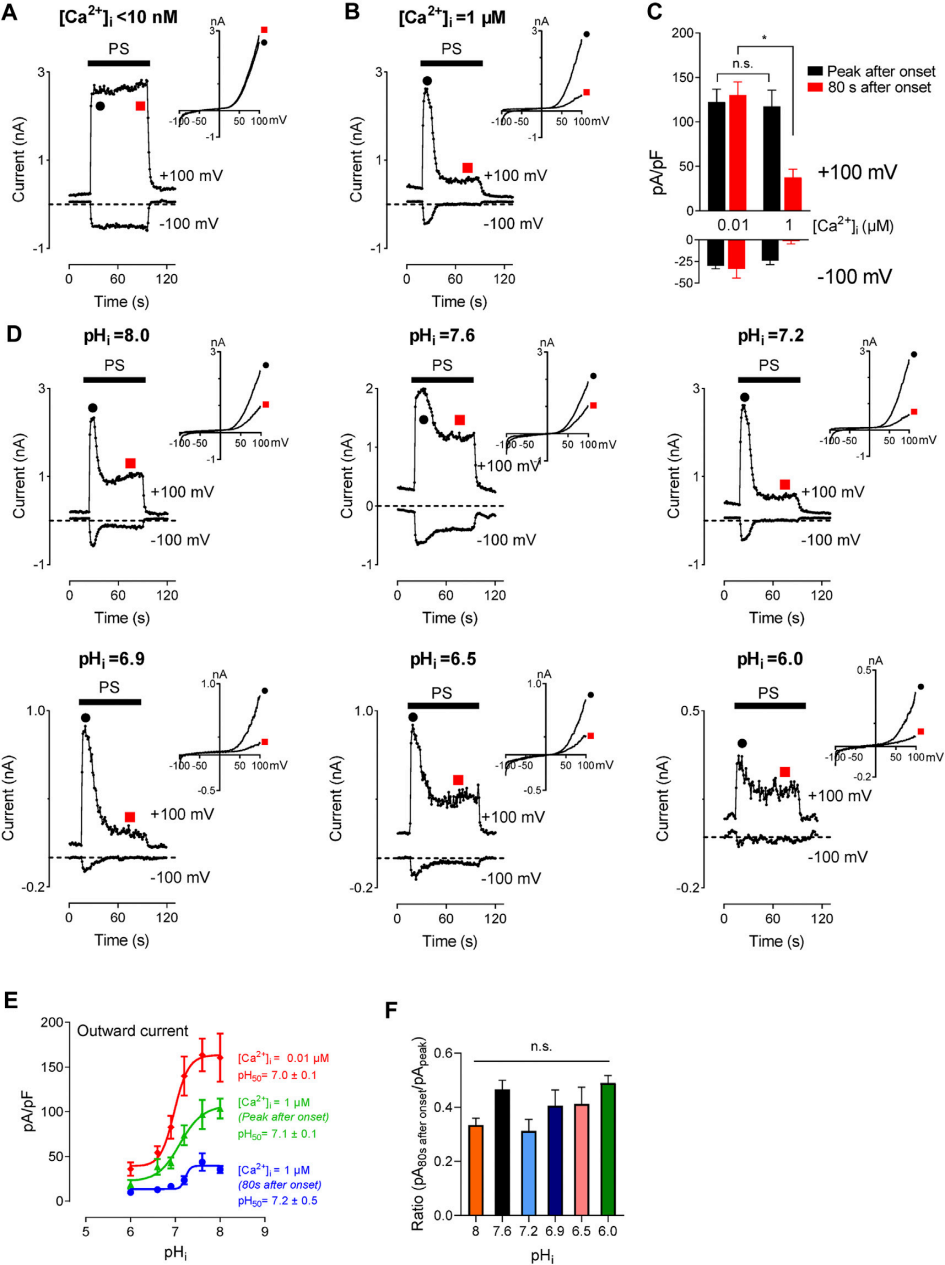
Inhibitory effect of intracellular high Ca^2+^ on TRPM3 activation by PS. **(A,B)** Time course of TRPM3 currents elicited by voltage ramps ranging −100 to +100 mV at the indicated [Ca^2+^]_i_ concentration. At high [Ca^2+^]_i_ (1 µM), TRPM3 is activated by PS initially (Black symbol) but runs down soon afterward (Red symbol). Whereas the TRPM3 current was sustained at the low [Ca^2+^]_i_ condition. Inserts show the representative recordings from the indicated points. **(C)** Mean current amplitude of TRPM3 at the indicated [Ca^2+^]_i_ (mean ± SEM; *n* = 10). * indicates *p* < 0.05 by unpaired Student’s t-test; “n.s.” indicates not statistically significant. **(D)** Time course and representative recordings of TRPM3 current with [Ca^2+^]_i_ (1 µM) at the indicated pH_i_ from pH_i_ 8.0 to pH_i_ 6.0. **(E)** pH concentration-dependence of TRPM3 activation by PS at the indicated [Ca^2+^]_i_. Green and blue symbols both represent high [Ca^2+^]_i_, while green represents currents elicited right after PS application (peak after onset), and blue represent currents remaining after inhibition of by high [Ca^2+^]_i_ (80 s after onset). All currents were normalized to the corresponding capacitance of the cell overexpressing hTRPM3. Each data point is the mean of 8–14 cells with the error bar showing SEM, at the indicated pH_i_. **(F)** Mean ratio of the peak current after onset to the current 80 s after onset (mean ± SEM, *n* = 8–14 cells). “n.s” indicates not statistically significant among groups by one-way ANOVA with Tukey’s post hoc multiple comparisons.

TRPM3 displays higher permeability for divalent cations than monovalent cations. For the splice variant TRPM3α2, 24% of total TRPM3 current is expected to result from Ca^2+^ (Przibilla et al., 2018), suggesting a large increase in [Ca^2+^]_i_ following TRPM3 activation. Multiple studies verified this effect showing an increase in [Ca^2+^]_i_ in a micromolar range following TRPM3 activation (Vriens et al., 2011; Straub et al., 2013; Przibilla et al., 2018). A recent study demonstrated that an increase in [Ca^2+^]_i_, independent of TRPM3 activity, inhibits TRPM3 in a calmodulin-dependent manner (Przibilla et al., 2018). This suggests a potential negative feedback mechanism that regulates a high increase in [Ca^2+^]_i_ resulting from TRPM3 activation. Studies conducted by others enlist [Ca^2+^]_i_ as a regulator of TRPM3 activity. In our study, we found that intracellular protons block TRPM3. Hence, we asked the question, how do these two regulatory mechanisms interact with each other? To find the effect of [Ca^2+^]_i_ on the concentration-dependent inhibition of TRPM3 by protons, we tested TRPM3 activity in two different [Ca^2+^]_i_ conditions, while providing a wide range of pH_i_. In addition to the inhibition of TRPM3 current by [Ca^2+^]_i_ observed by Przibilla (Przibilla et al., 2018), we observed an additionally delayed inhibition of TRPM3 current by Ca^2+^. For example, low [Ca^2+^]_i_ (<10 nM) did not show a delayed inhibition of TRPM3 current (red point, Figure 6A). Under 1 µM [Ca^2+^]_i_, the recorded current showed a further inhibition after initial activation and the residual current amplitudes were less than 50% of the initial activation (red point, Figures 6B,C). The inhibition was pH dependent (Figure 6D). However, the pH_i_ concentration-dependence of TRPM3 was not affected by [Ca^2+^]_i_, as they showed similar pH_50_ values (7.0 ± 0.1, n_H_ = 2.1; 7.1 ± 0.1, n_H_ = 1.4; 7.2 ± 0.5, n_H_ = 1.6) for low [Ca^2+^]_i_ and high Ca^2+^, (Figure 6E). It is noteworthy that despite having similar pH_50_ values, TRPM3 outward current densities were significantly decreased in high [Ca^2+^]_i_ conditions (Figure 6E). We also analyzed the ratio of delayed current intensities compared to the initial current intensities. We did not observe any significant difference between the ratios resulting from different pH_i_ (Figure 6F). Overall, these results indicate that, although [Ca^2+^]_i_ inhibits TRPM3, it does not affect the regulation of TRPM3 by intracellular protons.

### Molecular Mechanism Underlying Transient Receptor Potential Melastatin 3 Channel Inhibition by Protons

**FIGURE 7:**
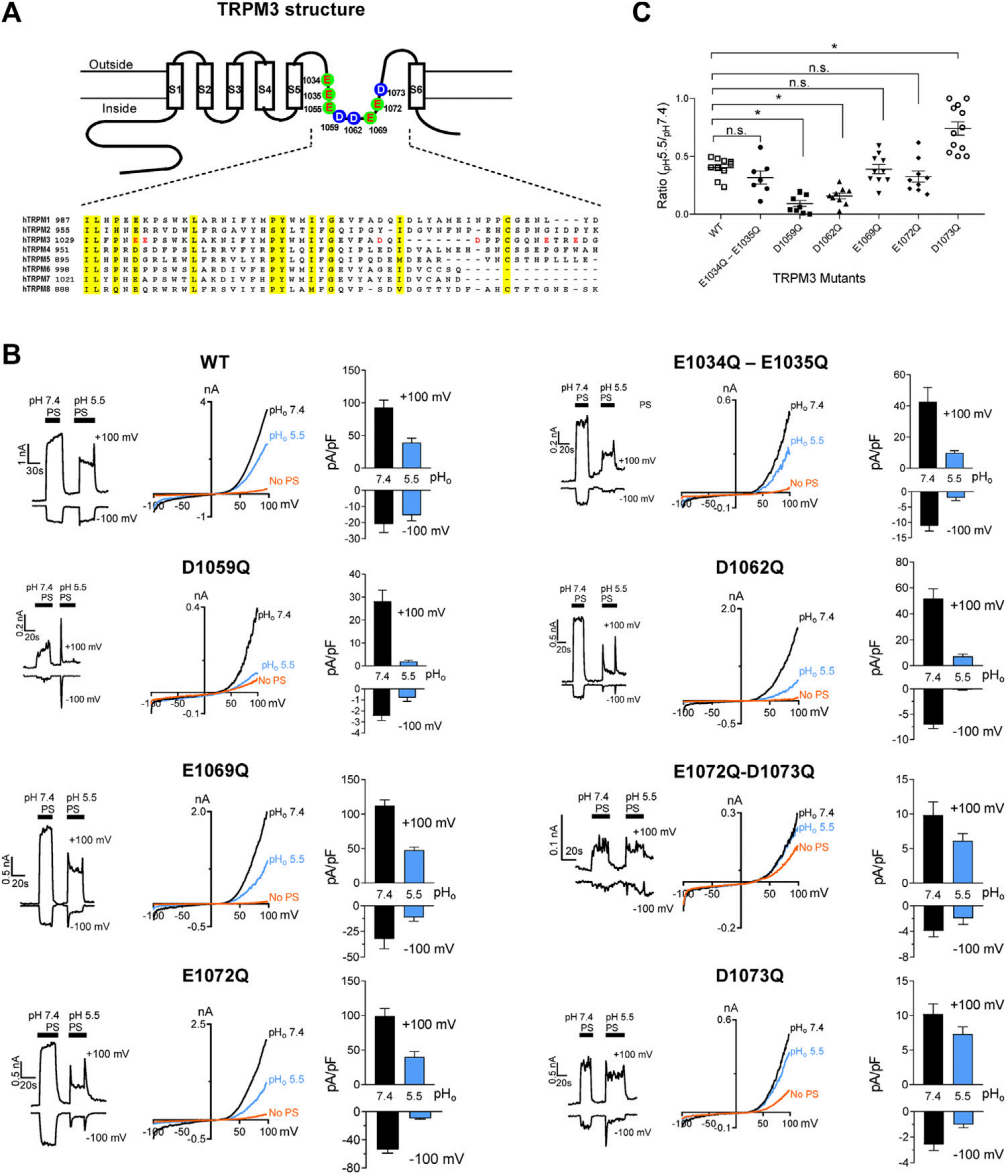
Changes in protons sensitivity of TRPM3 mutants. **(A)** Upper panel: schematic of TRPM3 structure and the substituted amino acid residues in the putative pore region of hTRPM3. Lower panel: sequence alignment of human TRPM channel pore regions. **(B)** Time course and representative recordings of TRPM3 mutants and wild-type currents elicited by voltage ramps ranging −100 to +100 mV, at the indicated pH_o_. Bar graphs show mean outward and inward current amplitudes at the indicated pH_o_ (mean ± SEM, *n* = 7–12 cells) Internal solution had a constant pH of 7.20 for all the recordings. **(C)** The ratio of outward current amplitudes at pH_o_ 5.5 and pH_o_ 7.4, of TRPM3 mutants and WT control. To obtain these ratios, the current elicited by +100 mV at pH_o_ 5.5 was divided by the current elicited by +100 mV at pH_o_ 7.4 of the same cell (*n* = 7–12 cells). *p* values, comparing each group with WT-TRPM3, * indicates *p* < 0.05 by unpaired Student’s t-test; “n.s.” indicates not statistically significant.

Glutamate, aspartate, histidine, and lysine residues are potential proton acceptors, especially glutamate and aspartate, which present negative charges (Zhou and Pang, 2018). To identify the amino acid residues of TRPM3 accountable for its sensitivity to pH_i_, we prepared hTRPM3-GFP mutant plasmids with mutations in the pore region. We selected all eight glutamate and aspartate amino acids, in the vestibule of the loop between S5 and S6 transmembrane domains (Figure 7A). These eight residues were either glutamate and aspartate amino acid and mutated to glutamine. We created two double mutants (E1034Q–E1035Q, E1072Q–D1073Q) and six single mutants (E1055Q, D1059Q, D1062Q, E1069Q, E1072Q, and D1073Q). We expressed these mutants in HEK-293 cells and recorded elicited currents in response to PS, while perfusing with physiological and low pH external solutions (Figure 7B). Except for E1055Q, which resulted in a non-functional ion-channel, all other mutants exhibited identical I-V relation to WT-hTRPM3, although most of the mutants showed markedly reduced current amplitudes (Figure 7B). We perfused cells expressing these mutants with external solutions of pH 7.4 and pH 5.5. The pH of 5.5 was selected as representative of low pH external solutions because at this pH, WT-TRPM3 currents were blocked significantly, yet sufficient activity remained for analysis. We compared the percent decrease in TRPM3 current from pH_o_ 7.4 to pH_o_ 5.5 for all the mutants and WT-TRPM3 (Figure 7C). The double mutant (E1034Q–E1035Q), E1069Q, and E1072Q showed similar sensitivity to protons when compared with the WT-TRPM3. Mutants D1059Q and D1062Q were found to be more sensitive to protons, as the reduction of whole-cell current due to low pH_o_ was increased to 93.9 and 84.1% in D1059Q and D1062Q, respectively, compared to 56.5% observed in WT-TRPM3. Mutant D1073Q showed significantly less sensitivity toward protons, as pH_o_ 5.5 reduced its current amplitude only by 25%. As summarized in Figure 7C, these results establish the amino acid residues D1059Q, D1062Q, and D1073Q as key determinants of proton sensitivity in TRPM3. Although it is unclear at this moment why mutating these residues produce variable effects (increased or decreased proton sensitivity), these data suggested that the pore vestibule of TRPM3 is critical for pH sensitivity. Further studies are required to delineate the underlying mechanism responsible for these variable effects.

## Discussion

Our data suggest that TRPM3 is a proton-permeable channel that is blocked by both extracellular and intracellular protons. We also demonstrated that the blocking effect of the intracellular protons could be reversed by decreasing intracellular protons concentrations, which indicates a reversible binding of protons to the intracellular amino acid residues. We identified several residues localized in the pore region that might be responsible for protons sensitivity of TRPM3. Finally, we demonstrated that low internal pH produces a downward shift of PS concentration-dependent activation of TRPM3 by reducing the amplitude of TRPM3-mediated currents at any given pH_o_. Overall, we report evidence for the regulatory role of protons on TRPM3 activation and the molecular mechanism responsible for it.

Acidification has modulatory effects on a variety of ion channels (discussed in the introduction) including TRP channels. Extracellular acidic pH modulates TRPV1 channel gating (Jordt et al., 2000; Ryu et al., 2003), stimulates TRPC4 and TRPC5, but inhibits TRPC6 (Semtner et al., 2007), TRPM5 (Liu et al., 2005), and TRPV5 (Yeh et al., 2003). Extracellular pH alsopotentiates TRPM6 and TRPM7 inward currents (Jiang et al., 2005; Li et al., 2006; Li et al., 2007). Intracellular protons, on the other hand, inhibit TRPM7 (Kozak et al., 2005), and block TRPM8 (Andersson et al., 2004). Here we demonstrate that PS-induced TRPM3 outward and inward currents are both inhibited by protons. Although both intracellular and extracellular acidic conditions inhibit TRPM3, there are differences between internal and external proton-induced inhibition. Extracellular protons cannot inhibit TRPM3 until pH reaches less than 6.0, but intracellular pH inhibited TRPM3 with pH_50_ value of 6.9. We demonstrated a very sharp inhibition by protons at an external pH below 6.0; however, this inhibition did not follow a typical concentration-response relationship. Conversely, internal protons efficiently blocked TRPM3 current and showed a concentration-response relationship. These results suggested that protons do not compete with PS for the same binding site of TRPM3 and that TRPM3 may contain proton-binding sites in the cytoplasmic domains. It is plausible that an increase in extracellular proton concentration causes protons influx through TRPM3 that enables protons to bind to its internal binding site to inhibit TRPM3. To rule out the effects of proton-activated endogenous Cl^−^ currents, we conducted all whole-cell recordings under very low intracellular and extracellular Cl^−^ concentrations. Indeed, our external and internal solutions contained 8 mM Cl^−^, but this proton concentration was not capable of activating endogenous anions channels in HEK-293 cells (Lambert and Oberwinkler, 2005).

We found that extracellular pH (<6.0) strongly blocked TRPM3 activities, which would indicate TRPM3 is regulated by extracellular acidic pH directly. However, the dose-dependent curve indictaed a low pH_50_. Reduced intracellular pH appeared to have clear pH concentration-dependent effects on TRPM3, which begs the question then how do external protons affect TRPM3 gating properties? The cell membrane must have a mechanism for the proton permeation and thus change the intracellular pH. Protons may cross the TRPM3 while it is open, although we cannot exclude the possibility that protons may permeate the cell membrane directly.

Our data suggest that protons directly cross TRPM3 when it is activated by PS. Consistently, no proton current was found without activating TRPM3, supporting the conclusion that TRPM3 is permeable to protons. We also studied the regulation of TRPM3 by Ca^2+^. Previous studies have shown that TRPM3 is a Ca^2+^ permeable ion channel (Grimm et al., 2003; Lee et al., 2003). TRPM3 channel activity strongly depends on intracellular Ca^2+^ (Przibilla et al., 2018). Along with the inhibition of TRPM3 current amplitude, we also found that Ca^2+^ accelerated the decay time of TRPM3. Increased [Ca^2+^]_i_ from minimum to 1 µM significantly reduced the plateau of the current. Becasue [Ca^2+^]_i_ is a key regulator of TRPM3 gating, we studied the interaction between [Ca^2+^]_i_ and protons. High [Ca^2+^]_i_ did not change the pH_50_ of the effects of intracellular protons on TRPM3 (Figure 6). Vice versa, decreased protons did not affect the Ca^2+^ inhibition on TRPM3 (Figure 6). Therefore, it appears unlikely that protons can inhibit TRPM3 channel activities by competing with [Ca^2+^]_i_ for a binding site on the cytoplasmic side.

Another well-studied TRPM channel that shows similar pH sensitivity is TRPM2. Extracellular acidic pH has been shown to inhibit TRPM2 with a pH_50_ = 6.5 (Starkus et al., 2010), 5.32 (Du et al., 2009), 4.7 (Yang et al., 2010), depending on the concentration of excellular and intracellular Ca^2+^ in the recording conditions. For example, Starkus et al. demonstrated that increasing extracellular Ca^2+^ counteracts the pH-induced inhibition (Starkus et al., 2010) and Du et al. demonstrated that increasing intracellular Ca^2+^ does not counteract the extracellular pH-induced inhibition (Du et al., 2009). In addition, similar to TRPM3, intracellular low pH also inhibits TRPM2 channels. Two groups have identified binding sites on the TRPM2 near the pore that might be responsible for the pH inhibition. Specifically, Lys952 and Asp1002 and several other residues in the outer vestibule were found to be important in mediating the extracellular pH inhibition whereas Asp933, is important in mediating the intracellular pH inhibition.

To determine the molecular mechanism by which intracellular protons inhibit TRPM3, we mutated all the titratable residues in the pore region between S5 and S6. Among most of the mutations of Asp and Glu residues, three residues-D1059, D1062, and D1073, which are predicted to reside in the pore region, strongly changed the proton sensitivities. We conclude these residues in the pore region could be the proton binding sites. Of course, other intracellular binding sites are also possible. For example, the C terminus of the S4-S5 linker is thought to be critical for changing the TRP channel’s pH_i_ sensitivity (Du et al., 2009). Whether intracellular protons change TRPM3 gating properties through these residues is still unknown, and thus it will be of interest to investigate whether acidic intracellular pH alters intracellular signaling pathways in future studies. It will also be of interest to investigate other potential proton binding sites that might interact with [Ca^2+^]_i_ near the intracellular mouth and act as the [Ca^2+^]_i_-activating site to regulate [Ca^2+^]_i_ mediated TRPM3 activation. Further investigation is required to test this hypothesis.

Our study might have significant physiological and pathophysiological implications since TRPM3 channels are expressed in adipocytes, pancreatic beta-cells, the kidney, eye, brain and in the pituitary gland. (Fonfria et al., 2006). Stimulation of endogenously expressed TRPM3 channels has been shown to trigger insulin secretion by insulinoma cells. Changes in extracellular pH are also known to affect glucose-stimulated insulin secretion (Hyder et al., 2001). Some reports suggest that during metabolic acidosis, insulin secretion is depressed (Bigner et al., 1996; Mak, 1998). Thus, it is possible that a low pH-mediated dampening of TRPM3 activity might contribute to the decreased insulin secretion observed in metabolic acidosis. If true, then modulating TRPM3 activity might be a potential future clinical application in treating acidosis induced pancreatic disorders. In addition, TRPM3 is required to induce release of calcitonin gene-related peptide (CGRP) (Held et al., 2015). In sensory neurons, the neuropeptide CGRP promotes neurogenic inflammation (Edvinsson et al., 1987). Thus, acidosis generated from infiltrating inflammatory cells might also inhibits TRPM3. Thus, although speculative, such a negative feedback mechanism of protons on regulation of TRPM3 might be beneficial in controlling these important cellular processes.

Collectively, we demonstrated that external and internal acidic pH show strong and state-dependent inhibition of the TRPM3 channels. Asp1073 residue in the vestibule of the channel pore is critical in modulating this inhibition. Given the physiological significance of TRPM3 in numerous cells, including pancreatic beta cells and sensory neurons, understanding TRPM3 gating by protons may generate new physiological and/or pathological insights.

## Data Availability Statement

The raw data supporting the conclusions of this article will be made available by the authors, without undue reservation.

## Author Contributions

JD conceived and supervised the project. JD, MS, and LX designed the experiments with input from Y-SL and LR, MS, and LX did most of the patch-clamp experiments. Y-SL and LR performed molecular biology experiments and oversaw the mutagenesis of TRPM3. MS and JD drafted the manuscript with input from all authors who contributed to finalizing the manuscript.

## Funding

JD is supported by the National Institutes of Mental Health (5R01 MH113986). LR is supported by the National Institutes of Health (R00HL119560, OT2OD023854, OT2OD026582).
