# Supplementary figures and images for: The Underlying Mechanism of Modulation of Transient Receptor Potential Melastatin 3 by protons
Source: Front Pharmacol. 2021 Feb 2;12:632711. doi: 10.3389/fphar.2021.632711 (PMC7884864; doi:10.3389/fphar.2021.632711)

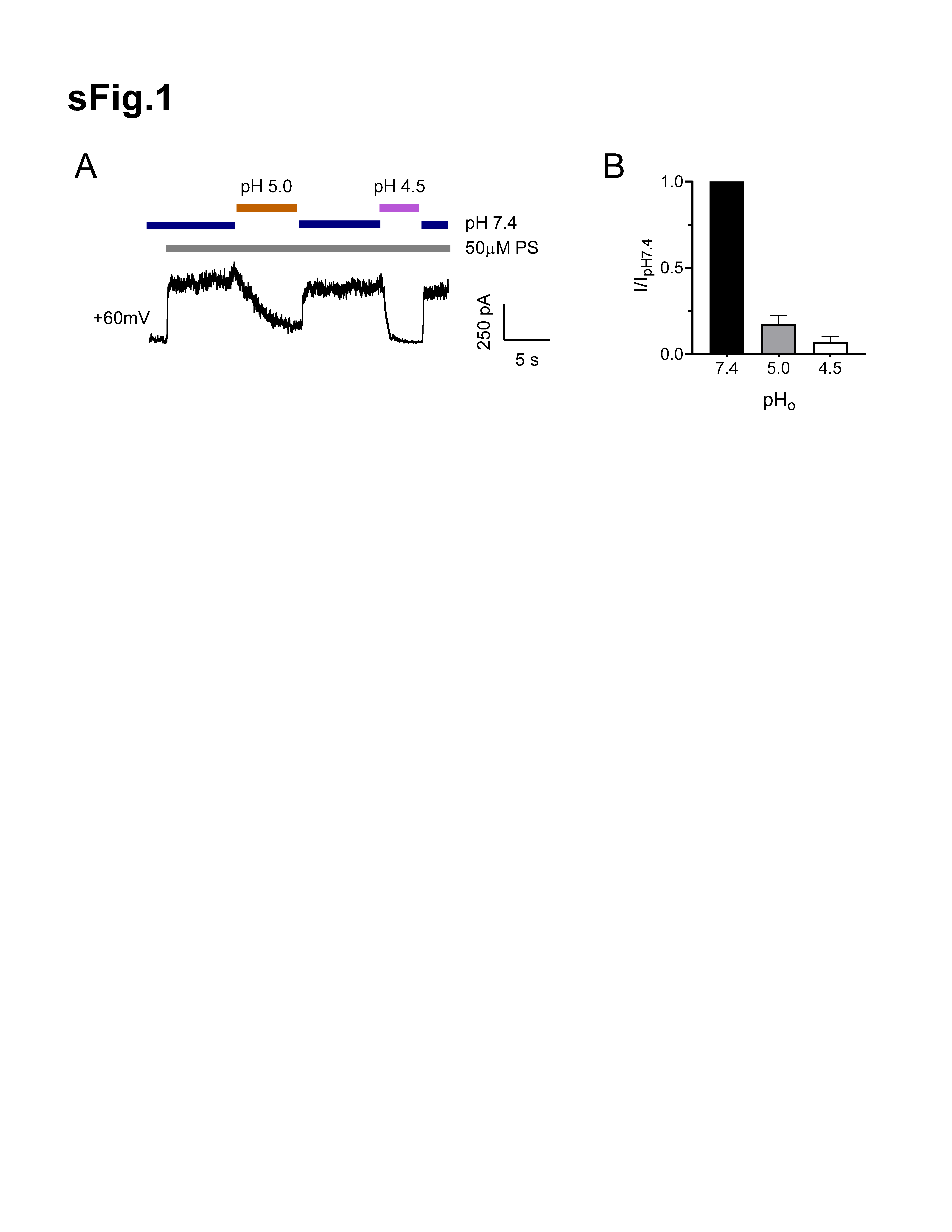

Supplement: Supplementary file 1 [file image1.tiff]

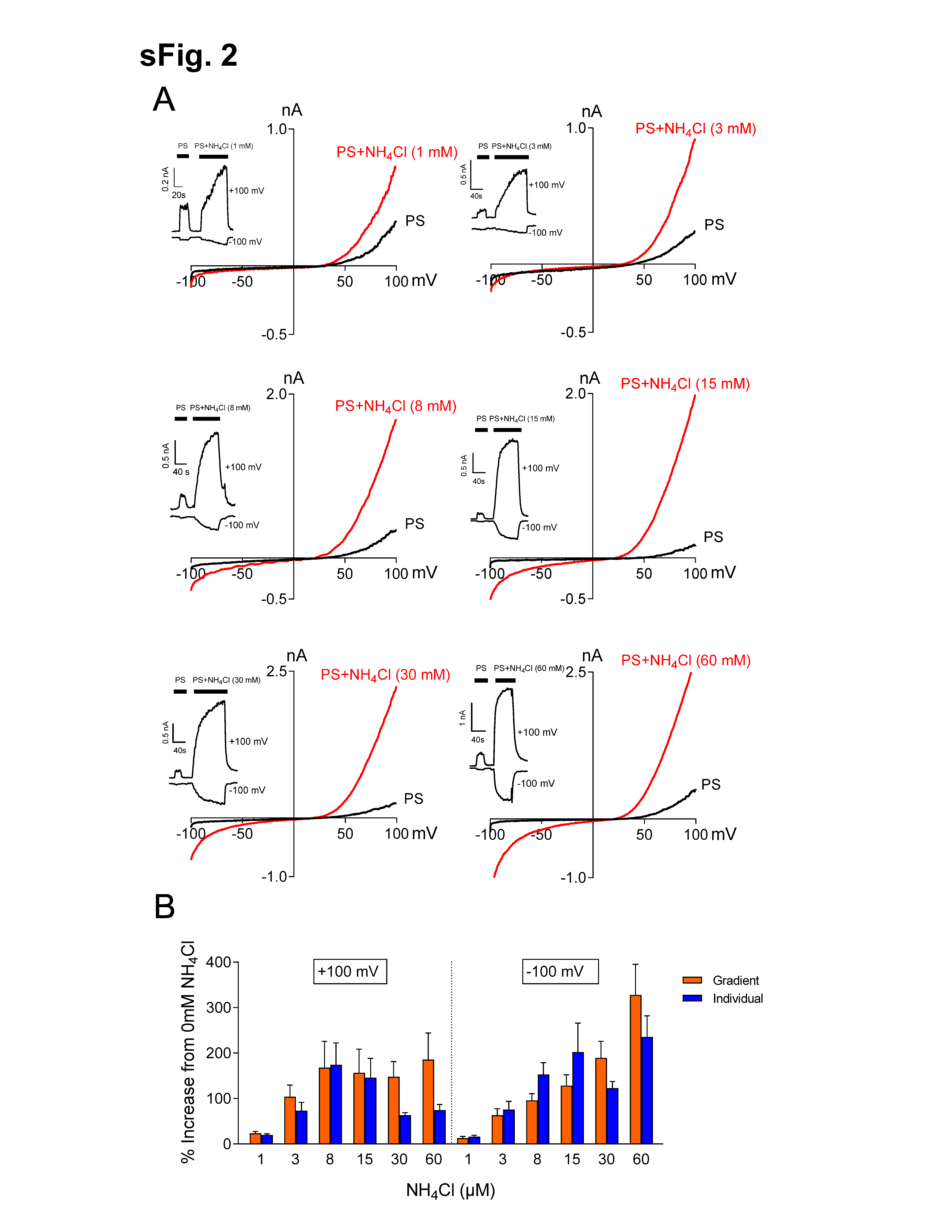

Supplement: Supplementary file 2 [file image2.tiff]
